# Trends in the Overall Incidence and Mortality of Lung Cancer: An Extensive Longitudinal Study Utilizing the CDC WONDER Database 1999–2020

**DOI:** 10.1111/1759-7714.70275

**Published:** 2026-05-19

**Authors:** Abdul Qahar Khan Yasinzai, Anaya Abdul Samad, Li Ziyang, Jaylyn Robinson, Muhammad Samsoor Zarak, Marjan Khan, Syed Ehsanullah, Teresa López Fernández, Nicolas L. Palaskas, Asad Ullah, Nagla Abdel Karim

**Affiliations:** ^1^ University of Florida Health Cancer Center Gainesville Florida USA; ^2^ Bolan Medical Complex Hospital Quetta Pakistan; ^3^ Texas Tech University Health Sciences Center Lubbock Texas USA; ^4^ Stanford Medicine Cancer Center Stanford California USA; ^5^ Marshfield Medical Center Marshfield Wisconsin USA; ^6^ La Paz University Hospital, IdipPAZ Research Institute Madrid Spain; ^7^ The University of Texas MD Anderson Cancer Center Houston Texas USA; ^8^ The George Washington University Washington DC USA

**Keywords:** databases (CDC), immunotherapy, lung neoplasms, mortality, regression analysis

## Abstract

**Background:**

Lung cancer remains a leading cause of global mortality despite declining rates. Since 2010, FDA‐approved immune checkpoint inhibitors (ICIs) have transformed treatment. This study investigates lung cancer incidence and mortality trends, emphasizing temporal associations with ICI integration and smoking patterns.

**Materials and Methods:**

Data (1999–2020) were extracted from the CDC WONDER database. Trends were analyzed using joinpoint regression to determine the annual percent change (APC).

**Results:**

Lung cancer incidence declined from 1999 to 2020, with the sharpest decrease post‐2018 (APC −6.90) compared to 2006–2018 (APC −1.78). Males saw steeper declines than females; however, American Indians/Alaska Natives showed an increasing trend (APC 3.96). Smoking prevalence decreased overall (1965–2018), though youth smoking rose between 1992 and 2004. By 2018, smoking was highest among American Indians/Alaska Natives (25.9%). Lung cancer mortality declined steadily since 1999, accelerating after 2014 (APC −4.10). Conversely, cardiovascular (CVS) mortality decline decelerated after 2015, stabilizing between 2018 and 2020 (APC 0.82; 95% CI −0.53 to 1.81).

**Conclusion:**

Lung cancer incidence and mortality declined significantly over two decades. The accelerated mortality declines post‐2014 coincide with the clinical integration of ICIs. The stabilization of CVS mortality after 2015 reflects a shift in the clinical profile, potentially related to competing risks and ICI‐related trends, warranting further investigation into whether population‐level CVS mortality is influenced by immunotherapy.

## Graphical abstract

Lung cancer incidence and mortality declined sharply post‐2014, coinciding with the integration of immune checkpoint inhibitors (ICIs) into standard care. Concurrently, the long‐term decline in cardiovascular mortality among these patients stabilized post‐2015, suggesting shifting clinical profiles and competing risks.

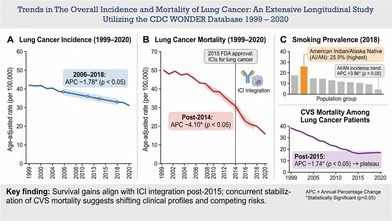

## 1 Introduction

Lung cancer is listed as one of the top causes of cancer‐related mortality worldwide by the World Health Organization (WHO). In 2022, there were nearly 2.5 million new cases and 1.8 million deaths globally [1]. The two major types of lung cancer are non‐small cell lung cancer (NSCLC), representing 80%–85% of lung cancer cases, and small cell lung cancer (SCLC), representing 10%–15% of lung cancer cases. Previous studies have reported declining trends in lung cancer incidence and mortality, largely attributed to reductions in smoking prevalence and the introduction of improved diagnostic and therapeutic strategies [2]. Nonetheless, variations in the magnitude and timing of these trends across demographic groups exist, and the specific contributions of declining risk factors compared with treatment improvements remain unclear.

Over the past decade, the introduction of immune checkpoint inhibitors (ICIs) has transformed the management of NSCLC, improving survival outcomes in patients with advanced disease [3]. In 2015, nivolumab (an anti–PD‐1 antibody) became the first ICI approved by the US Food and Drug Administration (FDA) for the treatment of advanced squamous NSCLC, marking the beginning of a new era in lung cancer therapy [4]. For lung cancer, the FDA‐approved ICIs fall into three main classes: programmed cell death protein 1 (PD‐1) inhibitors, programmed cell death‐ligand 1 (PD‐L1) inhibitors, and cytotoxic T lymphocyte‐associated antigen‐4 (CTLA‐4) inhibitors [5]. The PD‐1/PD‐1L pathway plays a critical role in cancer cells to escape from immunological surveillance.

Beyond oncologic outcomes, cardiovascular (CVS) health is a critical consideration in patients with lung cancer [6]. CVS disease is now recognized as a major non‐cancer cause of morbidity and mortality in this population [7]. Epidemiologic studies consistently show that about 23%–33% of lung cancer patients have a pre‐existing CVS diagnosis at the time of cancer diagnosis [8]. The most common conditions include congestive heart failure, myocardial infarction, cerebrovascular accident, and arrhythmias [9]. There is evidence that the burden of CVS comorbidity in lung cancer patients has increased over the past two decades, due to shared risk factors (smoking, age, hypertension, diabetes) and improved cancer survival [10].

The approval of immunotherapy has fundamentally changed the management of lung cancer; however, ICI‐associated cardiotoxicity is an important concern. Case reports and observational studies over the past decade have documented myocarditis, pericarditis, arrhythmias, and even fatal cardiac events associated with ICI treatment in lung cancer patients [11, 12, 13].

The goal of this study is to examine long‐term trends in lung and bronchial cancer incidence and mortality in the United States, with attention to demographic differences across sex, race, and ethnicity. In addition to overall cancer outcomes, the study examines CVS‐specific mortality among lung cancer patients to explore how population‐level shifts in comorbid conditions may coincide with evolving treatment landscapes. It also aims to explore potential factors underlying the recent decline in lung cancer mortality, including changes in smoking behavior and broader improvements in prevention, diagnosis, and treatment. The study seeks to provide a clearer understanding of progress in reducing the burden of lung cancer and to identify areas where disparities persist.

## 2 Materials and Methods

### 2.1 Data Source

Data was extracted from the Centers for Disease Control and Prevention (CDC) WONDER on September 15, 2023. Data were collected for the incidence of lung and bronchial cancer at the respective sites from 1999 to 2020. Additionally, data on mortality related to lung and bronchial cancer were categorized according to the International Classification of Diseases, Tenth Revision (ICD‐10). Consistent with the CDC WONDER data standard for the study period (1999–2020), which standardized data collected during the ICD‐9 to ICD‐10 transition, mortality for lung and bronchial cancer was identified using the ICD‐10 code C34 (Malignant neoplasm of bronchus and lung). Mortality due to both lung and bronchial cancer and diseases of the circulatory system (ICD‐10 codes I00–I99) were compiled together as multiple causes of death. This broad category was selected to capture the total CVS mortality burden, including ischemic heart disease, heart failure, and arrhythmias, providing a comprehensive view of circulatory‐related deaths in this population.

### 2.2 Ethics Statement

This study utilized de‐identified aggregate data from the CDC database, ensuring the privacy and confidentiality of individual patients. As a result, institutional review board approval was not required.

### 2.3 Statistical Analysis

To standardize the data and enable meaningful comparisons, we calculated crude rates of incidence per 100 000 individuals at risk using the 2000 US Standard Million population. The Joinpoint regression program (Version 5.0.2, National Cancer Institute; https://surveillance.cancer.gov/joinpoint/) was utilized to investigate annual percent change (APC) using the weighted Bayesian Information Criterion (BIC). The Empirical Quantile (EmpQ) method was used to execute the final model. The joinpoints were limited between 0 and 4. As this was a population‐level analysis using aggregate data, individual‐level treatment exposures were not available, and all observed trends represent temporal correlations rather than individual‐level causal effects.

## 3 Results

### 3.1 Incidence

**FIGURE 1:**
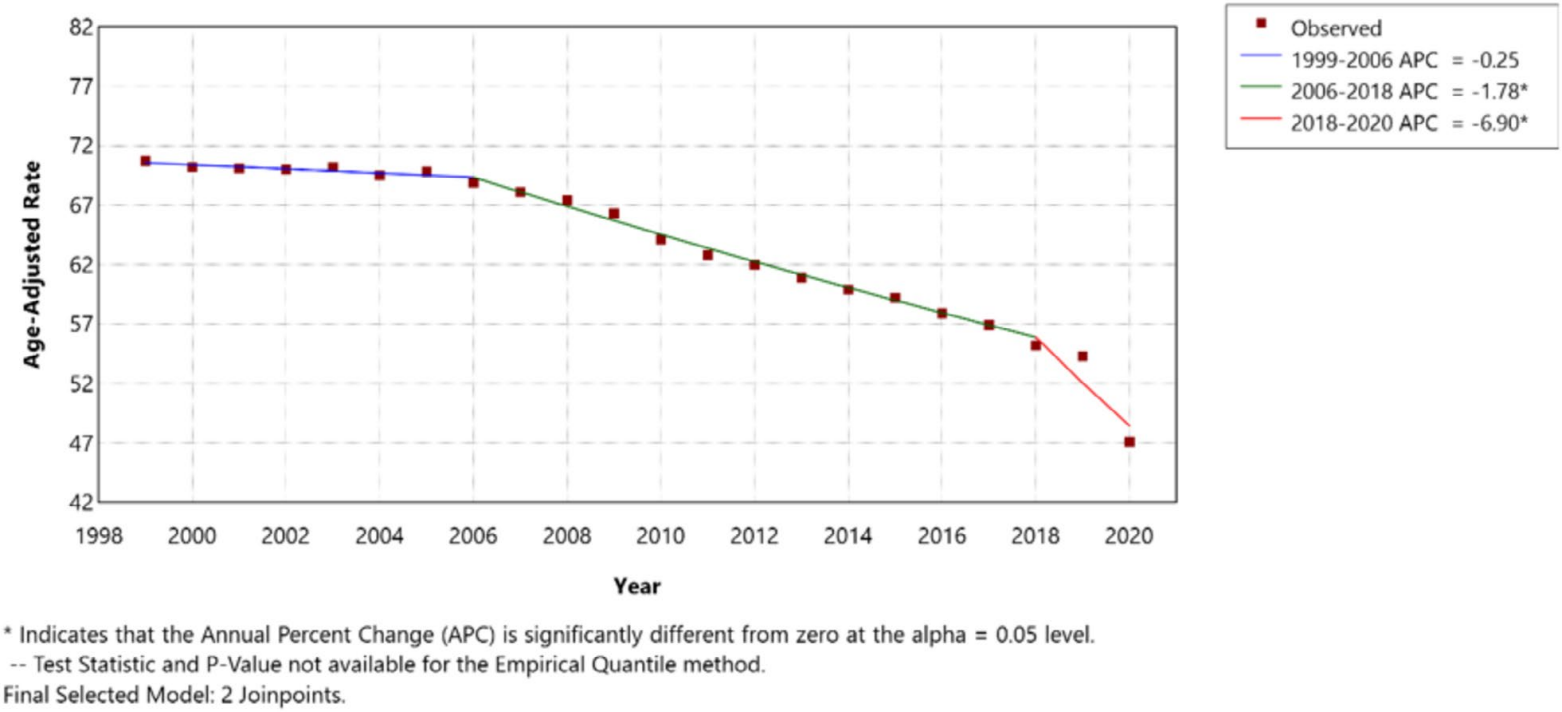
Annual percent change graph for the overall incidence of Lung and Bronchial cancer from 1999 to 2020 in the US population, abstracted from the CDC database.

**FIGURE 2:**
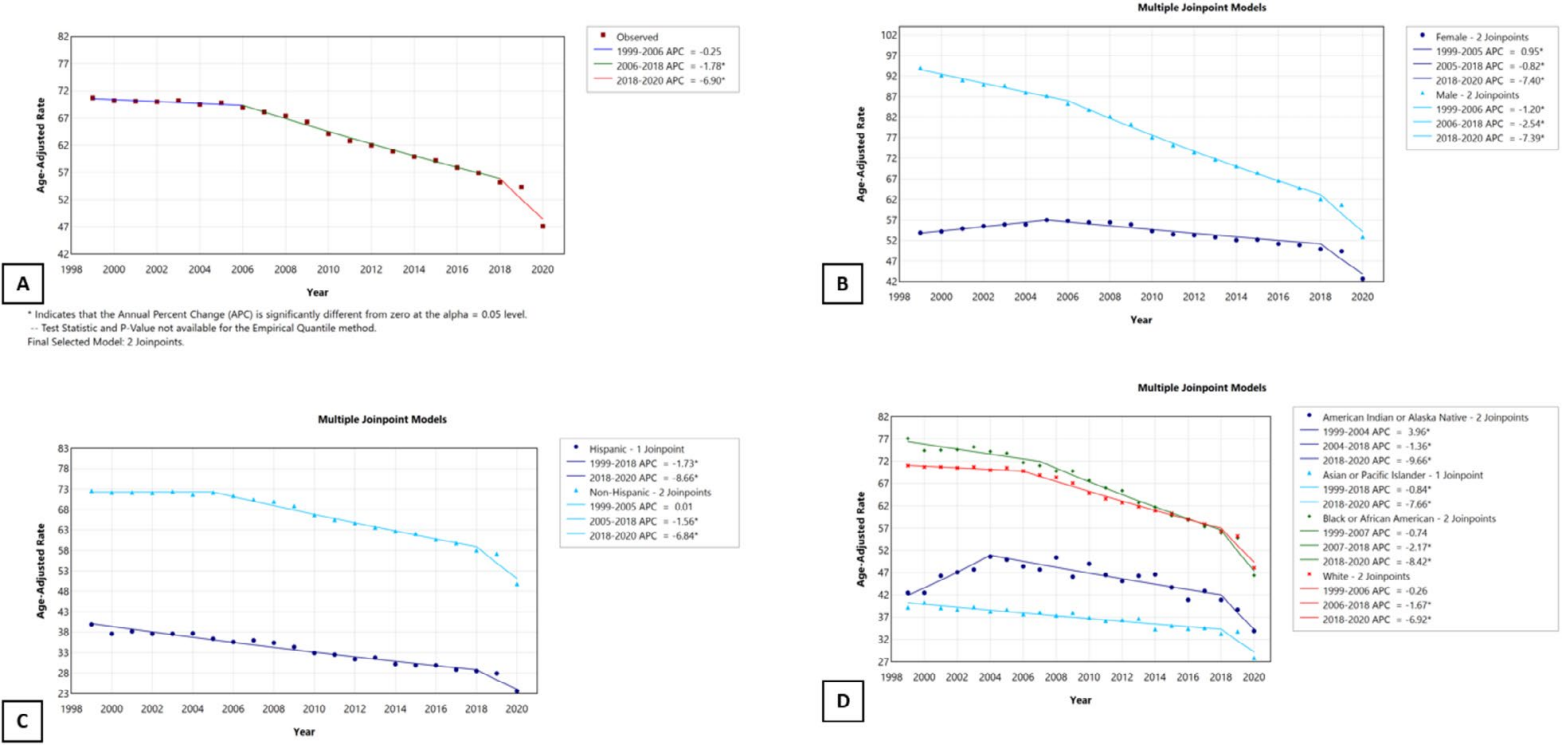
Annual percent change graph for the overall incidence of Lung and Bronchial cancer from 1999 to 2020 in the US population, abstracted from the CDC database. (A) Overall incidence, (B) incidence by sex, (C) incidence by Hispanic and non‐Hispanic ethnicities, (D) incidence by non‐Hispanic sub‐races.

The overall incidence of lung and bronchial cancer has been decreasing since 1999, but it saw a sharp, significant reduction from 2018 onward, with an APC of −6.90 compared to an APC of −1.78 from 2006 to 2018 and –0.25 from 1999 to 2006 (Figures 1 and 2). The incidence has been decreasing for both sexes; however, the male sex experienced a steeper reduction from 1999 to 2018 (males: APC –2.04 to −4.41; females: APC +0.96 to −3.68). Non‐Hispanics made a higher proportion of overall cases (95.8%), though the rate reduction is consistent between Hispanics and non‐Hispanics. When non‐Hispanic races were further stratified, the American Indians/Alaska Natives saw a significant increase in the incidence of lung and bronchial cancers by an APC of 3.96 compared to a consistent decline observed in other races.

### 3.2 Smoking Rate

**FIGURE 3:**
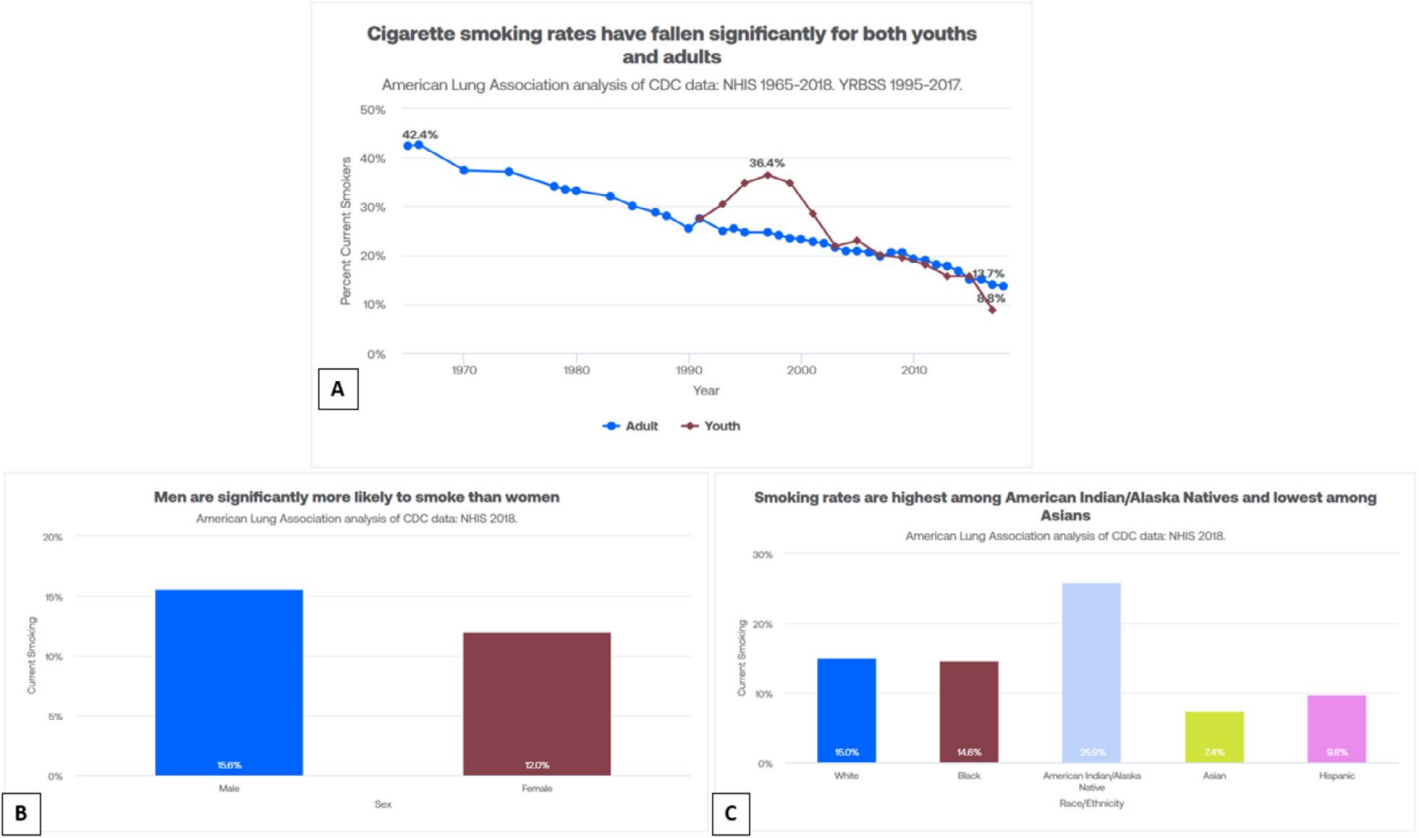
Cigarette smoking prevalence in the United States from 1965 to 2018 in the US population abstracted from the CDC database. (A) Smoking trends, (B) current (2018) smoking status by sex, (C) current (2018) smoking status by race.

In addition, smoking data demonstrated a consistent decline in cigarette smoking from 1965 to 2018 (Figure 3). The youth population had a significant increase in smoking from 1992 to 2004, but it then started declining along with adult smoking prevalence. Cigarette smoking proportions by sex from the CDC 2018 report suggest 25.8% of men and 14.1% of women use tobacco products. The smoking rate based on race in 2018 showed American Indians or Alaska Natives have the highest percentage of smokers, 25.9% followed by non‐Hispanic Whites, 15%. Blacks had a 14.6% prevalence; Hispanics had a 9.8% smoking prevalence, and Asians had the lowest smoking prevalence at 7.4%.

### 3.3 Mortality

**FIGURE 4:**
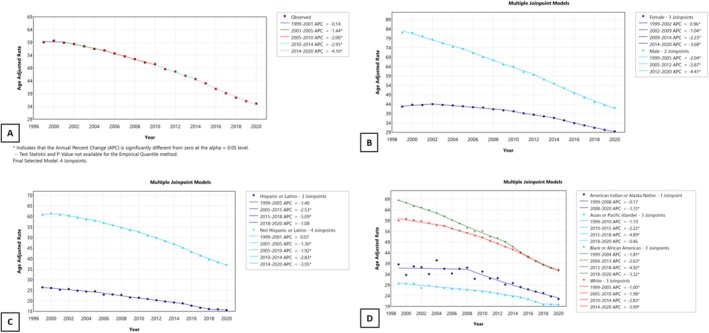
Annual percent change graph for the overall mortality associated with lung cancer from 1999 to 2020 in the US population, abstracted from the CDC database. (A) Overall mortality, (B) mortality by sex, (C) mortality by Hispanic and non‐Hispanic ethnicities, (D) mortality by non‐Hispanic sub‐races.

The overall mortality of lung cancer has been consistently declining since 1999, but the most significant decline was from 2014 onwards, with a significant APC of −4.10 (Figure 4). Mortality is declining for both males and females, but males had a steeper decline in mortality compared to females. Both non‐Hispanic and Hispanic ethnicities saw a significant decline in mortality, but both saw a significant reduction around 2014. Mortality in all non‐Hispanic races has been declining consistently since 1999.

**FIGURE 5:**
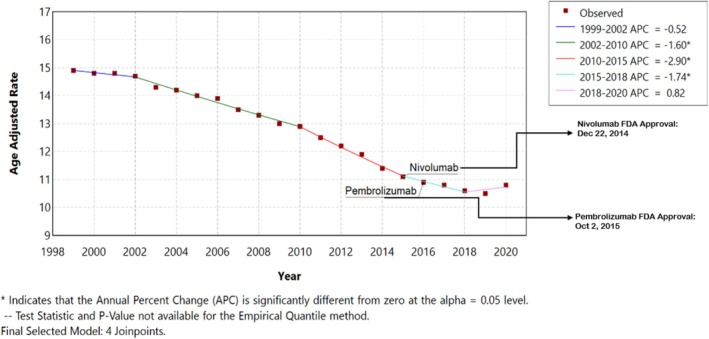
Annual percent change graph for the overall cardiovascular (CVS) mortality associated with lung cancer from 1999 to 2020 in the US population, abstracted from the CDC database.

The CVS mortality associated with lung cancer had an overall steady and consistent reduction until 2015 (Figure 5 and Table 1). From 2015 to 2018, the decline in CVS mortality slowed, with the APC changing from −2.90 to −1.74. However, from 2018 to 2020, the mortality trend plateaued, and the change was not statistically significant (APC = +0.82; 95% CI −0.53 to 1.81).

**TABLE 1:** Annual percent change and average age‐adjusted mortality rate for the overall incidence, overall mortality, and cardiovascular mortality associated with lung and bronchial cancer from 1999 to 2020 in the US population, abstracted from the CDC database.

Joint points	Segments	Timeline	APC	95% CI
INCIDENCE OF LUNG AND BRONCHIAL CANCER 1999–2020				
2	1	1999–2006	−0.2499	−0.8768 to 1.7288
	2	2006–2018	−1.7805^a^	−2.0979 to 1.5543
	3	2018–2020	−6.8965^a^	−8.2213 to −4.4218
Average AAMR	Full segment	1999–2020	1.7746^a^	−1.9337 to −1.5523
OVERALL MORTALITY OF LUNG AND BRONCHIAL CANCER 1999–2020				
4	1	1999–2001	−0.1364	−0.9181 to 0.7237
	2	2001–2005	−1.4388^a^	−2.1632 to −1.1725
	3	2005–2010	−2.0574^a^	−3.0850 to −1.5159
	4	2010–2014	−2.9497^a^	−4.8400 to −2.4669
	5	2014–2020	−4.1040^a^	−5.1322 to −3.1250
Average AAMR	Full segment	1999–2020	−2.5193^a^	−2.6269 to −2.4496
CARDIOVASCULAR MORTALITY IN LUNG AND BRONCHIAL CANCER 1999–2020				
4	1	1999–2002	−0.5197	−1.0094 to 0.3496
	2	2002–2010	−1.6039^a^	−2.0513 to −1.3922
	3	2010–2015	−2.8984^a^	−3.5531 to −1.1643
	4	2015–2018	−1.7406^a^	−3.1416 to −1.2872
	5	2018–2020	0.8232	−0.5374 to 1.8169
Average AAMR	Full segment	1999–2020	−1.5513^a^	−1.6425 to −1.4802

Abbreviations: AAMR, average age‐adjusted mortality rate; APC, annual percent change; CI, confidence interval.

^a^ Indicates significant change.

## 4 Discussion

Our study shows a consistent decline in the incidence of lung cancer, smoking, and mortality. Most importantly, the survival gain was very considerable from 2014 to 2020, coinciding with the regulatory approval and clinical rollout of ICIs. In 2015, the Food and Drug Administration first approved Nivolumab for treating lung cancer, and several ICIs were approved in the following years [14, 15, 16]. As seen in Figure 4, lung cancer mortality declined steadily, with a sharper decrease observed after 2014. While this timing coincides with the FDA approval of ICIs, the long‐term decline in smoking prevalence among both adults and youths has also been a major contributor to reductions in lung cancer incidence and mortality. Our findings align with the reported benefits of ICIs in phase III clinical trials [17]. For example, the PACIFIC trial demonstrated a significant overall survival improvement with durvalumab in unresectable stage III NSCLC [18]. Similarly, KEYNOTE and CheckMate trials have shown survival gains in advanced NSCLC [19, 20]. However, when comparing these results with population‐level statistics, the observed decline in lung cancer mortality is more gradual. Although clinical trials demonstrate efficacy, the translation of this benefit to a population‐wide survival advantage depends on factors such as eligibility, access, and the uptake of ICIs in real‐world settings [17]. Nevertheless, there are some remaining challenges, including a large number of “nonresponders” and ways to convert “nonresponders” to “responders” [21, 22].

More importantly, the chronic immune‐related adverse events (irAEs) of ICIs have become more recognized and continue to persist in long‐term follow‐up, affecting up to 43.2% of patients [23]. The potential pathophysiological mechanisms behind ICI‐related toxicities described in clinical literature include shared T‐cell receptors between malignant and non‐malignant tissues [24]. While most chronic irAEs are classified as endocrine or rheumatological, CVS involvement is increasingly reported and clinically important [24, 25, 26].

Cardiac immune‐related adverse events (irAEs) are uncommon but clinically significant complications of ICI therapy in lung cancer, with myocarditis, pericarditis, and arrhythmias being the most frequently reported manifestations [27]. Myocarditis carries a high mortality risk, and arrhythmias are the most prevalent cardiac irAE [28]. Risk is further elevated in patients with comorbidities such as diabetes, hypertension, or hyperlipidemia [29], and appears higher with ICI monotherapy or ICI combined with chemotherapy compared to chemotherapy alone [30]. Importantly, thoracic radiotherapy acts synergistically with ICIs to exacerbate cardiotoxicity [31], particularly in patients with prior immune‐related events [32].

Cardiotoxicity is one of the serious chronic irAEs that needs to be addressed with close attention. ICIs can cause cardiac conditions, including but not limited to myocarditis, arrhythmias, myocardial infarction, and accelerated pre‐existing heart disorders. Risk factors could be categorized as treatment‐related factors, such as dual or combined immunotherapy, previous CVS disease with myocardial injury, previous autoimmune disease, and tumor‐related factors. The first case reports of fatal myocarditis associated with ICIs were described by Johnson et al. in 2016, following initial doses of combined nivolumab and ipilimumab therapy [26].

In our study, Figure 4 indicates that CVS mortality in lung cancer patients declined steadily until approximately 2015. Following this period, the previous downward trend plateaued. While this deceleration in CVS mortality coincides with the FDA approval and clinical integration of ICIs, such as Nivolumab and Pembrolizumab, it did not reach statistical significance as an increase [15, 16]. Given the nature of population‐level data, these findings do not establish a direct causal link to immunotherapy‐related cardiotoxicity but rather highlight a shift in the mortality profile that warrants further clinical investigation. The nuances of these population trends are further highlighted when compared to existing literature. Howlader et al. reported continued declines in incidence‐based NSCLC mortality, reflecting therapeutic advances. This discrepancy may partly relate to differences in cause‐of‐death attribution within national datasets, where lung cancer‐related and CVS deaths may be misclassified or over‐attributed [33].

As survival improves with immunotherapy, competing risks such as pre‐existing CVS disease become more apparent. This “competing risk” phenomenon suggests that as lung cancer‐specific mortality declines and patients survive longer, they are naturally at higher risk for age‐related CVS events. Consequently, the observed stabilization in CVS mortality may reflect this shift in survival dynamics rather than being solely attributable to drug‐related toxicity.

While myocarditis is a well‐recognized but rare irAE, it cannot fully explain the observed plateau in CVS mortality. Emerging evidence suggests that ICIs may accelerate atherosclerotic plaque formation and destabilization, thereby increasing the risk of ischemic heart disease, which remains the leading cause of CVS death [34]. While this mechanism could theoretically contribute to the observed plateau in CVS mortality, the ecological nature of our data precludes a definitive link to ICI exposure at the individual level. In addition, other cardiac irAEs, including arrhythmias, pericardial disease, and heart failure, may also contribute, particularly in patients with pre‐existing comorbidities or those receiving thoracic radiotherapy [35].

We report the current trend of lung cancer incidence in the United States by race and sex, quantifying the annual percent change of trends with joinpoint regression. The incidence of lung cancer has continued to decline with the significant drop in smoking rate [36, 37]. Our study observed that women had a relatively higher incidence of lung cancer compared to men, but this incidence has also shown a significant decline over time. This aligns with Jemal et al., who suggested that sex differences in smoking behaviors may not fully explain such trends. Part of the decline in men could be due to reduced synergistic occupational exposures [38].

In addition to gender disparities among smokers, racial disparities continue to exist, with more male smokers and American Indians/Alaska Natives having the highest percentage of smokers in the year of 2018. However, while American Indians or Alaska Natives ranked highest in the number of smokers in 2018, it's important to take into account racial differences in lung cancer incidence. At low cigarette consumption, Native Hawaiians and African Americans have twice as high lung cancer incidence as Japanese and Latinos, and the difference could be related to smoking and quitting behaviors [39, 40, 41].

## 5 Limitations

There are several limitations in the study regarding the database itself. Our data on mortality were based on death certificates. Clinical information relating to medical treatment, vital signs, and hemoglobin levels could not be collected to support the direct relationship between immunotherapy and cardiac‐related death in lung cancer patients. We also could not differentiate whether the toxicity is directly attributed to ICI or due to anthracyclines, which are known to be cardiotoxic. Additionally, from the study design perspective, our study is observational; as a result, causality cannot be determined with certainty. Our dataset is limited in individual‐level data on medical treatments, such as specific ICI drug exposure or duration. Therefore, we cannot definitively link the observed mortality trends to immunotherapy at the patient level. Our methodological definition of CVS mortality utilized the broad ICD‐10 grouping (I00–I99). While this provides a comprehensive overview of the CVS disease burden, a limitation of this approach is the inability to distinguish between specific subtypes of cardiotoxicity, such as ischemic heart disease versus non‐ischemic manifestations (e.g., myocarditis or arrhythmias), which should be a focus of future granular studies.

## 6 Conclusion

The overall incidence and mortality of lung cancer are decreasing during the study time period. The considerable decline in mortality after 2014 is likely suggestive of ICI integration into the standard of care. The stabilization of CVS mortality trends after 2015 reflects an associative shift in the clinical profile of lung cancer patients. The CVS mortality changes observed during different timelines may be suggestive of treatment paradigm shifts, but causal inference cannot be confirmed. Future research may explore whether ICI truly can impact CVS mortality on a population level, and whether further optimizing the dosage may improve survival and reduce toxicities in lung cancer patients.

## Author Contributions

**Abdul Qahar Khan Yasinzai:** conceptualization, methodology, data abstraction, analysis, software, writing – original draft preparation. **Anaya Abdul Samad:** investigation, literature review, writing – review and editing. **Li Ziyang:** literature review, writing – original draft preparation. **Jaylyn Robinson:** literature review, writing – original draft preparation. **Muhammad Samsoor Zarak:** investigation, writing – review and editing. **Marjan Khan:** investigation, writing – review and editing. **Syed Ehsanullah:** investigation, writing – review and editing. **Teresa López Fernández:** investigation, validation, writing – review and editing. **Nicolas L. Palaskas:** investigation, validation, writing – review and editing. **Asad Ullah:** methodology, literature review, writing – original draft preparation. **Nagla Abdel Karim:** conceptualization, supervision, validation, project administration, writing – review and editing.

## Funding

The authors have nothing to report.

## Conflicts of Interest

The authors declare no conflicts of interest.

## Data Availability

The data underlying this article are available in the CDC WONDER database (https://wonder.cdc.gov/). The datasets were derived from publicly available sources.
